# A Research Protocol for Implementation and Evaluation of a Patient-Focused eHealth Intervention for Chronic Kidney Disease

**DOI:** 10.1007/s43477-022-00038-3

**Published:** 2022-01-30

**Authors:** Maoliosa Donald, Heather Beanlands, Sharon Straus, Lori Harwood, Gwen Herrington, Blair Waldvogel, Maria Delgado, Dwight Sparkes, Paul Watson, Meghan Elliott, Kerry McBrien, Aminu Bello, Brenda Hemmelgarn

**Affiliations:** 1 Department of Medicine, University of Calgary, HSC G239, 3330 Hospital Drive NW, Calgary, AB T2N 4N1 Canada; 2 Daphne Cockwell School of Nursing, Ryerson University, Toronto, ON Canada; 3 Department of Medicine, University of Toronto, Toronto, ON Canada; 4 London Health Sciences Centre, London, ON Canada; 5 Can-SOLVE CKD Patient Partner, Vancouver, BC Canada; 6 Department of Family Medicine, University of Calgary, Calgary, AB Canada; 7 Department of Medicine, University of Alberta, Edmonton, AB Canada

**Keywords:** Implementation science, eHealth, Chronic kidney disease, Quality implementation framework, Person-centered care

## Abstract

Self-management in chronic kidney disease (CKD) can slow disease progression; however, there are few tools available to support patients with early CKD. *My Kidneys My Health* is a patient-focused electronic health (eHealth) self-management tool developed by patients and caregivers. This study will investigate the implementation of *My Kidneys My Health* across primary care and general nephrology clinics. The study aims to: (1) identify and address barriers and facilitators that may impact implementation and sustainability of the website into routine clinical care; (2) evaluate implementation quality to inform spread and scale-up. We will conduct a multi-stage approach using qualitative methods, guided by the Quality Implementation Framework and using a qualitative content analysis approach. First, we will identify perceived barriers and facilitators to implementation and considerations for sustainability through interviews with clinicians, based on the Readiness Thinking Tool and the Long Term Success Tool. Analysis will be guided by the Consolidated Framework for Implementation Research and the Theoretical Domains Framework. Appropriate implementation strategies will be identified using the Expert Recommendations for Implementing Change compilation, and implementation plans will be developed based on Proctor’s recommendations and the Action, Actor, Context, Target, Time framework. Finally, we will explore implementation quality guided by the RE-AIM framework. There is limited literature describing systematic approaches to implementing and sustaining patient-focused self-management tools into clinical care, in addition to employing tailored implementation strategies to promote adoption and sustainability. We aim to generate insights on how *My Kidneys My Health* can be integrated into clinical care and how to sustain use of patient-centric eHealth tools in clinical settings on a larger scale.

**Supplementary Information:**

The online version contains supplementary material available at 10.1007/s43477-022-00038-3.

## Introduction

Chronic kidney disease is a progressive condition, often characterized by multi-morbidity that impacts treatment and management. To slow disease progression, persons with chronic kidney disease are required to balance medical management of their disease and other chronic conditions (e.g., diabetes, cardiovascular disease, depression) with the demands of daily life (Tonelli et al., 2015). Self-management is a promising approach to slow disease progression and improve health outcomes important to patients. It aims to facilitate an individual’s ability to make positive changes by focusing on illness needs, activating resources, and developing coping skills to live with the biopsychosocial demands of the disease and related comorbidities (Richard & Shea, 2011).

International chronic kidney disease research priority setting activities have identified a need to develop optimal strategies to support patients to self-manage their chronic kidney disease and related comorbidities (Hemmelgarn et al., 2016; National Kidney Foundation, 2017; Tong et al., 2015). Our research team (i.e., patient partners [patients and caregivers], clinicians, researchers, and policy makers), supported by a pan-Canadian, patient-oriented research (POR) initiative (Can-SOLVE CKD Network), is investigating opportunities to enhance chronic kidney disease self-management support for patients (Levin et al., 2018). We are conducting a multi-phased project examining strategies and tools for chronic kidney disease self-management most valued by patients and those who care for them. Important to these individuals are pertinent, tailored, timely information and resources to live well with chronic kidney disease, especially for those in early stages of the disease process (i.e., not on dialysis) or recently diagnosed (Donald et al., 2019a, 2019b). An electronic health (eHealth) platform has potential to address these priorities.

While there are numerous eHealth self-management interventions available for patients with chronic kidney disease, they typically focus on later stages of chronic kidney disease and lack key characteristics important in providing person-centered care (Donald et al., 2018; Smekal et al., 2019). First, they have been predominantly designed by health care professionals without patient input (Bonner et al., 2018; Donald et al., 2018; Shen et al., 2019). Second, only a few self-management interventions have been theory-informed for the planning, design, and evaluation of the intervention (Donald et al., 2018; Shen et al., 2019). Finally, there is sparse literature in regard to planning, measuring, and reporting implementation and sustainability efforts for chronic kidney disease self-management eHealth interventions (Ghimire et al., 2015; Shen et al., 2019).

To address these knowledge gaps, we undertook a theory-informed, person-centered approach to develop an eHealth tool, the *My Kidneys My Health* website, that is concordant with patients’ values, needs, and preferences for chronic kidney disease self-management support (Donald et al., 2021a). The focus of this paper is to describe the process of implementation and evaluation of the *My Kidneys My Health* website.

The key objectives are to identify and address provider and organizational barriers and facilitators that may impact implementation and sustainability of the *My Kidneys My Health* website into routine clinical care for individuals with chronic kidney disease; and explore implementation quality within primary care and general nephrology clinics to understand and inform spread and scale-up in these settings.

## Design and Methods

We will conduct a multi-stage approach using qualitative methods to undertake the implementation (i.e., integration into practice) and evaluation of the *My Kidneys My Health* website guided by the quality implementation framework (QIF) (Meyers et al., 2012). Reporting of data will be guided by the Consolidated Criteria for Reporting Qualitative Research guidelines (Tong et al., 2007).

### Website Overview

The evidence-informed *My Kidneys My Health* website (www.mykidneysmyhealth.com) was co-developed using a systematic, user-centered design process previously described (Donald et al., 2021a). The results of our explanatory sequential mixed-methods feasibility study demonstrated that the *My Kidneys My Health* website had high user acceptance, with a statistically significant increase in perceived self-efficacy sub-scale, *gaining information* (Donald et al., 2021b). It is an open-access, patient-centered web application, accessible by computer or mobile device. *My Kidneys My Health* was designed based on a conceptual framework identifying optimal design features for eHealth interventions (i.e., interactivity between social context and support, direct contact with the intervention, tailored information, and self-management) (Morrison et al., 2012), patient preferences (Donald et al., 2019b), and behavior change theory (Baay et al., 2019). The website supports self-management by “informing” (chronic kidney disease related information); “activating” (prompts/tools to encourage action to manage chronic kidney disease and enhance quality of life); and “collaborating” (links/tools that lead to interaction and engagement with health care professionals and peers) (Donald et al., 2021a; Gagliardi et al., 2015). Specific elements of the website include interactive tools; personalized food list and health care professionals question list; and multimedia education and resources. While the website is targeted for patients, clinicians play a key role in offering and engaging patients through this resource. For example, the website can be used opportunistically, or at the point of care, when individuals are diagnosed with chronic kidney disease or share a concern related to supporting chronic kidney disease self-management.

### Quality Implementation Framework

**Fig. 1:**
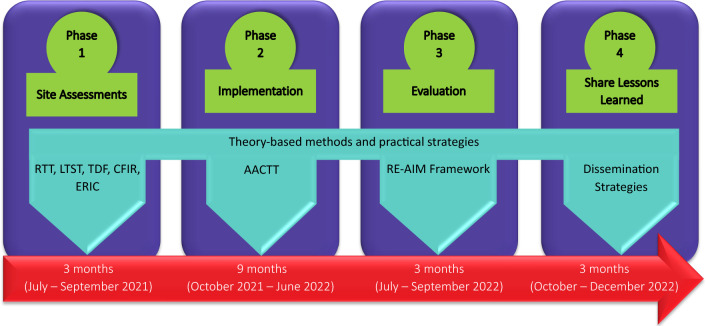
Quality Improvement Framework guiding implementation and sustainability of My Kidneys My Health website. *Note AACTT* action, actor, context, target, time, *CFIR* consolidated framework for implementation research, *ERIC* expert recommendations for implementing change, *LTST* long term success tool, *RE-AIM* reach, effectiveness, adoption, implementation, and maintenance, *RTT* readiness thinking tool, *TDF* theoretical domains framework

Research supports the use of implementation science in improving the quality of implementation and ultimately achieving desired patient outcomes (Durlak & DuPre, 2008). The Quality Implementation Framework (QIF) is an implementation process model providing mechanisms and strategies for successful implementation of an intervention (Blanchard et al., 2017). It is based on 25 implementation frameworks and consists of six themes with 14 steps, grouped into four phases that are practical and intuitive to our clinical context (see Fig. 1) (Meyers et al., 2012). The four phases entail site assessments, implementation, evaluation, and sharing lessons learned.

### System Engagement and Implementation Team

**Fig. 2:**
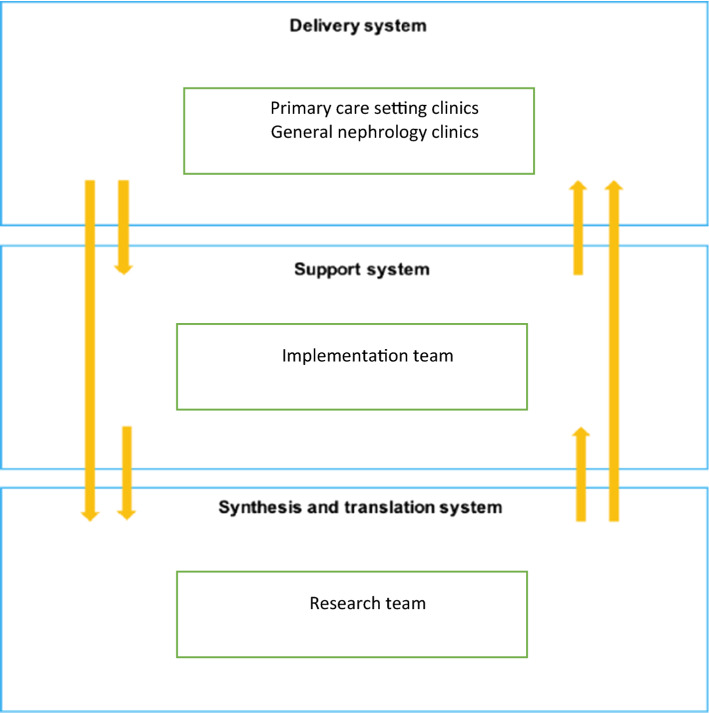
System engagement—applying the implementation system framework

Guided by the Knowledge-to-Action Framework, key stakeholders (i.e., patient partners, clinicians, policy makers, and organizations involved in the care of individuals with chronic kidney disease) were engaged in the co-design of the evidence-informed *My Kidneys My Health* website (Donald et al., 2021a). We will continue to work with these stakeholders to achieve quality implementation, applying the Interactive Systems Framework (ISF) to identify stakeholder roles and to situate the QIF within the clinical and research context. The ISF considers who is in the delivery system (clinicians in the primary care setting and general nephrology clinics), support system (implementation team), and synthesis and translation system (research team) (see Fig. 2) (Wandersman et al., 2012). The delivery system is responsible for carrying out the activities needed to integrate the website into clinical practice. The implementation team will oversee and support implementation efforts of the *My Kidneys My Health* website at the sites throughout all stages of implementation. It is recommended that members of the team have knowledge about the intervention, as well as implementation experience (Fixsen et al., 2007). Specifically, team members will help inform, prepare, and support the sites to effectively integrate the website into practice. The team members will include: an implementation lead with experience in the co-design of the website and knowledge in implementation science; a support coach (project coordinator) and patient partner with knowledge of the intervention components; two clinicians (a nephrologist and a primary care physician with experience in managing patients with chronic disease, in addition to understanding primary care organizational functioning); and a knowledge broker with skills in knowledge translation. Our research team is considered part of the synthesis and translation system, co-creating the *My Kidneys My Health* website.

### Setting

For the purposes of this study, we plan to implement the *My Kidneys My Health* website in two distinct settings within the Canadian context: primary care and general nephrology. Care for patients with chronic kidney disease varies depending on etiology of chronic kidney disease, disease severity, and presence of comorbidities. For example, patients with early stage, stable, or slowly progressive chronic kidney disease with other chronic conditions are usually cared for by primary care physicians. Furthermore, primary care physicians play a key role in identification and management of patients with chronic kidney disease, in addition to coordinating care with specialists such as nephrologists (Levin et al., 2013).

Working with our current stakeholders and networks in Alberta (i.e., Alberta Kidney Care, Primary Care Networks, Medicine Strategic Clinical Network, Primary Health Care Integration Network) we will identify six primary care clinics and two general nephrology clinics. The networks comprise patients, clinicians, researchers, and decision-makers who are knowledgeable about specific areas of health, with goals to achieve better outcomes and better value for Albertans (Noseworthy et al., 2015). We will approach sites that see patients with chronic kidney disease and associated comorbidities. We will strive for diversity with respect to geographic clinic location (i.e., rural/urban), services provided (i.e., team-based/individual PCP) and clinics that serve diverse populations (e.g., to gain a better understanding of how context affects implementation and sustainability).

### Proposed Phases and Processes

The following activities will be completed during the four phases: site assessments, implementation, evaluation, and learning from the implementation experience (see Fig. 1). An overview of the theory-based methods supporting this work is provided (see Table 1).

**Table 1:** Overview of theory-based methods

Method	Study application	References
Qualitative improvement framework (QIF)	QIF will be used to guide the overall study phases	Meyers et al. (2012)
Interactive systems framework (ISF)	ISF will assist us identifying relevant stakeholders and their roles within the following systems: delivery, support, and synthesis and translation	Wandersman et al. (2012)
Readiness thinking tool (RTT)	RTT and LTST will inform open-ended questions for the pre-implementation interview guide	Wandersman et al. (2012)
Long term success tool (LTST)		Lennox et al. (2017)
Theoretical domains framework (TDF)	TDF and CFIR will be used to understand individual and system barriers and facilitators associated with implementation	Atkins et al. (2017)
Consolidated framework for implementation research (CFIR)		Damschroder et al. (2009)
Expert recommendations for implementing change (ERIC)	ERIC to identify relevant implementation strategies	Powell et al. (2015)
Action, actor, context, target, time (AACTT) framework	AACTT will direct operationalizing the implementation strategies	Presseau et al. (2019)
Evidence-based system for innovation support (EBSIS) framework	EBSIS will inform the types of implementation supports needed by individuals within the delivery system	Wandersman et al. (2012)
RE-AIM framework	RE-AIM will be used to evaluate the adoption and implementation, in addition to understanding elements for sustained use of the intervention	RE-AIM (2021)

#### Phase 1: Site Assessments

The aim of this phase is to identify perceived barriers and facilitators (determinants) to implementation (i.e., integration into practice by offering and/or engaging patients with the website), as well as considerations for sustainability of the *My Kidneys My Health* website for use within the primary care and general nephrology settings by assessing readiness and sustainability. Identified determinants will inform our selection of appropriate implementation strategies. The findings from this phase will be used to inform the implementation plan for the clinical sites.

Using a purposive sampling strategy at the eight clinics, we will invite staff (e.g., primary care physicians, nephrologists, allied health) who provide direct clinical care and education to patients with chronic kidney disease, as well as clinic administrators who oversee site operations with key decision-making roles as the potential participant group. There are no exclusion criteria. To obtain maximum variation in professional roles, genders, and practice settings we will set quotas for the minimum number of participants that should be represented in each of the specified groups. We anticipate approximately 25 to 30 interviews based on previous similar studies (Eldh et al., 2020; Greer et al., 2019).

Telephone interviews will be 30-min, and the semi-structured interview guide will include a series of questions that are based on the Readiness Thinking Tool (RTT) (Wandersman et al., 2012) and the Long Term Success Tool (LTST) (see Supplementary File 1—pre-implementation interview guide). The RTT consists of multiple constructs (e.g., motivation and capacity) that can be applied across various levels, including the individual, team, and organization (Wandersman et al., 2012). The LTST is relevant for the healthcare setting and consists of 12 key factors to identifying risks and prompt actions to increase chances of sustainability over time (Lennox et al., 2017).

The interviews will be conducted in the summer and fall of 2021 by the implementation lead (MD) and/or implementation support team member, both with qualitative research experience. Participants will also complete a short, structured survey describing their role and practice arrangements (e.g., professional background, years of experience, clinical site structure, familiarity with self-management resources for chronic kidney disease, etc.) (see Supplementary File 2—demographic questionnaire). Interviews will be digitally recorded and uploaded to a secure server. A trained transcriptionist will provide a verbatim, deidentified transcript (i.e., removal of participant and clinic names) of the interview. A research staff member will review transcripts for accuracy and confidentiality before uploading to NVivo Version 12 (QRS International, Inc.) for storing and organizing the data.

The analysis will focus on understanding individual and system barriers and facilitators that are associated with implementing and sustaining the *My Kidneys My Health* website as a self-management support tool for patients, in primary care and general nephrology clinic settings. We will conduct a qualitative directed content analysis using a deductive coding approach to coding guided by two frameworks (Hsieh & Shannon, 2005). One, applying the Consolidated Framework for Implementation Research (CFIR) (Damschroder et al., 2009), comprised five major domains: the intervention, inner and outer setting in which it is implemented, the individuals involved in the implementation, and the process by which implementation is accomplished (Damschroder et al., 2009). Two, using the Theoretical Domains Framework (TDF), a comprehensive, theory-informed framework to identify individual determinants of behavior (Michie et al., 2014). The TDF can be used to assess implementation problems and support implementation planning (Atkins et al., 2017). Two research team members will independently familiarize themselves with the data, then review all transcripts line-by-line to identify sections of text that reflect the domains of the CFIR and TDF. After independently coding the first 3 interviews, the two members will meet and compare the labels they have applied and agree on the set of codes to apply to all subsequent transcripts. They will then chart the data by creating a matrix that presents codes categorized to individual, system, and intervention barriers and facilitators, along with illustrative quotations (Gale et al., 2013). The analysis will be iterative and as a final step the first author (MD) will review the coding schema with the original transcripts. For the interpretation of the data the following questions will be considered: (1) How do inner factors (e.g., organizational culture, leadership) influence implementation and sustainment?; (2) How do outer context factors (e.g., financial) influence implementation and sustainment?; (3) What are the perceptions of clinicians and decision-makers to implementation and sustainment?; (4) To what extent do factors and perceptions vary by site (e.g., organization and geographic)?

To address barriers and enhance facilitators to implementation we will identify relevant implementation strategies (tools, activities, and/or actions) that will support adoption of the website. Acknowledging that different strategies can be used at multiple levels within the delivery system (i.e., clinic, team, individual health care professionals) we will refer to the Expert Recommendations for Implementing Change (ERIC) compilation (Powell et al., 2015). The ERIC taxonomy has the following categories: (1) engage consumers, (2) use evaluative and iterative strategies, (3) change infrastructure, (4) adapt and tailor to the context, (5) develop stakeholder interrelationships, (6) use financial strategies, (7) support clinicians, (8) provide interactive assistance, and (9) train and educate stakeholders with 73 discrete implementation strategies, which can be combined to form a multifaceted strategy (Waltz et al., 2015).

#### Phase 2: Implementation

The aim of this phase is to design a detailed implementation plan that includes describing the implementation strategies, operationalizing these strategies, and designating implementation supports needed to deliver the website.

**Fig. 3:**
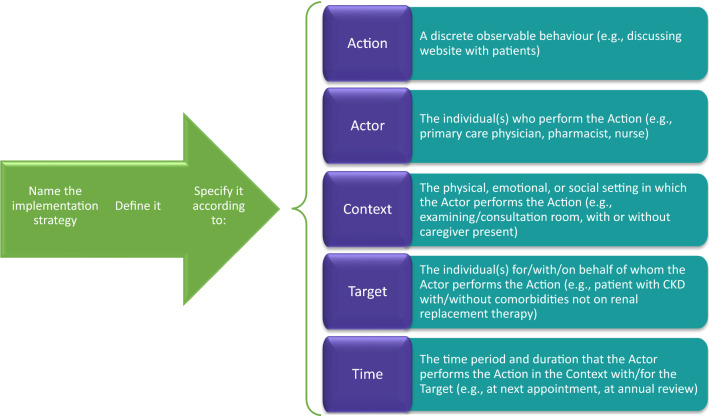
Implementation plan based on proctor’s guidance for specifying implementation strategies and AACTT framework. *Note AACTT* action, actor, context, target, time

We will describe and operationalize the implementation strategies based on Proctor’s recommendations for specifying and reporting implementation strategies (Proctor et al., 2013) and the Action, Actor, Context, Target, Time framework (Presseau et al., 2019). Figure 3 describes the implementation strategy, specifically who needs to do what differently, when, where, and for whom. We will also consider what types of implementation supports will be needed for the clinician/administrator to offer and engage patients with chronic kidney disease with the website. Based on the Evidence-Based System for Innovation Support framework for implementing innovations with quality, these supports can include tools, training, technical assistance, and quality assurance to address initial needs and problems that arise during the implementation period (Wandersman et al., 2012). For example, these supports may include check-in calls and site visits, or identifying a “site implementer” to provide ongoing support. In addition, we will develop a process to monitor and provide supportive feedback to sites based on their needs.

#### Phase 3: Evaluation

The aim of this phase is to explore implementation quality guided by the RE-AIM framework (reach, effectiveness, adoption, implementation, and maintenance) to understand the adoption, implementation, and sustained use of the *My Kidneys My Health* website. The RE-AIM framework is an evaluation framework that measures success of an intervention implemented within a given context (Glasgow et al., 2019). For this component, we will focus on the following elements: reach, adoption, implementation, and maintenance.

We will conduct 30-min telephone interviews 9 months post-implementation using a purposive sampling strategy at the eight clinics, similar to pre-implementation. We will invite staff that both had an interest or lack thereof in the use of the *My Kidneys My Health* website. A semi-structured interview guide will include a series of questions that are based on the RE-AIM Qualitative Guide (see Supplementary File 3—post-implementation interview guide) (Holtrop et al., 2018; RE-AIM, 2021). Reach will provide us with information about health care professionals’ level of interest in the intervention and in engaging patients to use the website (e.g., attitudes toward addressing chronic kidney disease self-management with patients). Adoption will be assessed by understanding how health care professionals engaged patients (e.g., what informal criteria they used for selecting which patients) and how they integrated the *My Kidneys My Health* website into their management for patients with chronic kidney disease. We will explore the implementation dimension by investigating to what extent the website is implemented as intended (e.g., consistency of promoting website; challenges to embedding into clinical care). We will investigate the dimension of maintenance by exploring the extent to which the website will be sustained in routine care going forward (e.g., intended sustained use). In addition, participants will complete a short, structured survey describing their role and practice arrangements (see Supplementary File 2).

Interview data will be analyzed using a deductive qualitative directed content analysis approach similar to the activities outlined in Phase 1. Data will be examined based on the CFIR and TDF domains to provide structure for the factors identified and to fully understand the underpinnings behind the results (Damschroder et al., 2009). Data that does not fall within the framework will be captured to ensure ideas are not being missed. Interpretation of the findings will focus on implementation successes, failures, and when, where and what types of adaptations are needed to overcome future implementation issues and ensure sustainability.

#### Phase 4: Learning from the Implementation Experience

The aim of this phase is to share learnings from our research findings to broader communities (e.g., patients and families, clinicians, researchers, decision-makers) through public engagement forums, webinars, publications, and conference presentations with the intention to improve future implementation efforts of patient-focused eHealth tools in similar settings. In addition, we anticipate that we will build stronger inter-organizational relationships based on the co-planning, implementation, and evaluation of the *My Kidneys My Health* website. As part of this project, we will work appropriate partners to ensure that evaluation is a continuous process within their settings.

## Discussion

Implementation science uses established theories and frameworks to understand contextual and other factors related to the use of an innovation (Bauer & Kirchner, 2020). To successfully implement a patient-focused eHealth intervention tailored for patients in primary care and general nephrology we need to understand the potential implementation problems, desired implementation behaviors, and contextual factors, and identify the appropriate evidence-based implementation strategies. To the best of our knowledge, this is the first implementation science study of the integration of a patient-focused eHealth tool tailored for patients in primary care and general nephrology context. We are proposing to test a “formula for success” where we are implementing an evidence-informed intervention (*My Kidneys My Health* self-management website), with quality implementation (QIF), in an enabling context (primary care and general nephrology clinics). The goal of this study is to generate knowledge about implementing and sustaining an eHealth tool in the primary care and general nephrology setting. Applying the QIF to guide this work will support the achievement of implementation outcomes based on a strategic approach to identifying implementation strategies for the sites.

The knowledge gained from this study will strengthen the implementation and sustainment of eHealth self-management interventions in chronic kidney disease care and clinical care settings by identifying barriers and facilitators, as well as implementation strategies appropriate to real-world clinical contexts. Anticipated impacts of this study include moving toward enhanced chronic kidney disease self-management and the application of a standard approach to the development and customization of transferrable implementation strategies and tools for use in different care settings. It is anticipated that the use of QIF activities will support successful implementation with the intention to promote self-management strategies and enhance desired health outcomes, in addition to informing how to implement patient-centric eHealth tools in clinical settings on a larger scale.

There are limitations to this study. This study is limited to eight participating sites and the health care professionals within these clinical settings. The participants may not be representative of health care professionals in other contexts. A potential limitation of the evaluation process may be recall bias when obtaining interview data after the implementation period (post 9 months) as adherence may fluctuate.

However, we believe the use and integration of several different theoretical perspectives is an approach that will extend the literature in implementation science and help identify how such theoretical perspectives can be combined to offer a comprehensive approach to implementing evidence-informed interventions into other settings.

## Supplementary Information

Below is the link to the electronic supplementary material.

Supplementary file1 (PDF 157 kb)

Supplementary file2 (PDF 142 kb)

Supplementary file3 (PDF 148 kb)

## Data Availability

The datasets used and/or analyzed during the current study will be available from the corresponding author on reasonable request.
