# Supplementary material for: A Research Protocol for Implementation and Evaluation of a Patient-Focused eHealth Intervention for Chronic Kidney Disease
Source: Glob Implement Res Appl. 2022 Jan 30;2(1):85–94. doi: 10.1007/s43477-022-00038-3 (PMC8938369; doi:10.1007/s43477-022-00038-3)
Supplement: Supplementary file 1 — Supplementary file1 (PDF 157 kb) [file 43477_2022_38_MOESM1_ESM.pdf]

Supplementary File 1

Interview Guide Pre-Implementation

1. **Fit (compatibility, tension for change, adaptations, and relative priority)**
   a) How does integrating the My Kidneys My Health website into your practice (i.e., the "intervention") fit with your personal values and norms?
   b) How does the intervention fit with clinic's values and norms?
   c) How does the intervention fit with existing workflow and current practices in your clinic?
      - What are the likely issues or complications that may arise?
   d) What types of high-priority initiatives or activities related to self-management are already happening in your clinic? (CKD-related or non-CKD related)
      - What is the priority of implementing the website relative to other initiatives that are happening now?
      - How will the implementation conflict with other priorities?
      - How will the implementation help achieve (or relieve pressure related to) these priorities?
   e) How might the My Kidneys My Health website replace or complement a current program or process?
      - Which programs and/or processes? If replacing, why? If complementing, how?
   f) Does the website need to be modified in any way to fit clinic setting and/or patient population? If yes, how?
   g) What are your thoughts about the need for this website?
      - Does it meet the needs of patients? Providers? How/why not?

1. **Available Resources/Support**
   a) Tell me about the resources/supports that you currently have in place to administer the website (i.e., promote to patients/caregivers and support use).
      - Are the resources sufficient? Do you feel as though you/your team has sufficient knowledge and skills for implementation?
      - Who will support the clinicians/staff to administer the website?
      - What shortfalls, if any, might you anticipate?
      - What resources/support would help to administer the website?

2. **Leadership Engagement**

a) What kind of support or actions can you expect from senior leaders in your clinic to help improve the likelihood of a successful implementation? [*note: assumes you are speaking with frontline staff, adjust according to the target audience*]

- Who are these leaders [*not looking to identify specific individuals rather the positions these leaders hold*]? How do attitudes of different leaders towards change vary?
- Are they aware of the intent to implement the intervention?
- What kind of support can you expect going forward? Can you provide specific examples?
- What types of barriers might they create?

3. Culture of Improvement and Learning

a) To what extent do you feel that you can try new things to improve the care of your patients/work processes?

b) To what extent do you feel the clinic can try new things?

4. Communication and Collaboration

a) With respect to the working relationships between staff members:

- Do staff work together collaboratively? Please explain.
- Have they established trusting relationships? Please explain.
- How is information (e.g., new initiatives, accomplishments, challenges, staff changes) communicated?
- Do you feel there is consistent and clear communication? Please explain.

5. Sustainability

a) Do you feel other staff should be involved in implementation? If so, which staff?

b) How will new staff become familiar with the intervention?

c) How would you measure success of using the website?

- Are there ways of measuring that currently?
- What would those look like?

d) What measures would you be interested in to assess impact of this intervention?
