# Supplementary material for: A Research Protocol for Implementation and Evaluation of a Patient-Focused eHealth Intervention for Chronic Kidney Disease
Source: Glob Implement Res Appl. 2022 Jan 30;2(1):85–94. doi: 10.1007/s43477-022-00038-3 (PMC8938369; doi:10.1007/s43477-022-00038-3)
Supplement: Supplementary file 2 — Supplementary file2 (PDF 142 kb) [file 43477_2022_38_MOESM2_ESM.pdf]

Supplementary File 2

Demographic Questionnaire

1. Where do you work?
   - General nephrology clinic
   - Primary care practice

2. Where is the clinical practice located?
   - Small rural population centres (<1,000 to 29,999)
   - Medium rural population centres (30,000 to 99,000)
   - Large urban population centres (100,000 to 499,999)
   - Large metropolitan urban centres (> 500,000)

3. What is your current primary role?
   - Primary care physician
   - Nephrologist
   - Nurse
   - Nurse practitioner
   - Dietitian
   - Pharmacist
   - Social worker
   - Administrator
   - Other ____________

4. What is your current age?
   - Under 40
   - 40 to 64 years
   - 65 years or older
   - Prefer not to answer

5. What is your level of post-secondary education?
   - Diploma or certificate
   - College or university
   - Graduate school
   - Other____________
   - Prefer not to answer

6. What is your current employment status?
   - Full-time
   - Part-time (please specify, FTE: _____)
   - Casual
   - Other ___________

7. Do you have any of the following team members within your practice (check all that apply)?
   - Primary care physician

- Nephrologist
- Nurse
- Nurse practitioner
- Dietitian
- Pharmacist
- Social worker
- Administrator
- Other ____________

8. Which term best describes your gender identity?
    - Female
    - Male
    - Or, please specify your gender identity: ________
    - Prefer not to answer

9. How many years have you been in clinical practice?
    - 0 to 5 years
    - 6 to 10 years
    - 11 to 15 years
    - 16+ years
    - Prefer not to answer

10. Are you familiar with any of the following CKD self-management online resources for patients (check all that apply)?
    - My Kidneys My Health website
    - Kidney Foundation of Canada website
    - Other _________
