# Supplementary material for: A Research Protocol for Implementation and Evaluation of a Patient-Focused eHealth Intervention for Chronic Kidney Disease
Source: Glob Implement Res Appl. 2022 Jan 30;2(1):85–94. doi: 10.1007/s43477-022-00038-3 (PMC8938369; doi:10.1007/s43477-022-00038-3)
Supplement: Supplementary file 3 — Supplementary file3 (PDF 148 kb) [file 43477_2022_38_MOESM3_ESM.pdf]

Supplementary File 3

Interview Guide Post-Implementation

1. **Reach**
   a) How did you hear about the My Kidneys My website?
      - Through the study (completed pre-implementation interview); staff (during implementation)

   b) How interested were you in promoting the website to your patients?
   c) What made you interested in promoting, or not interested, promoting the website to your patients?
      - Website content, access, etc.; resources/time

   d) What made it easier to promote the website?
   e) What made it harder to promote the website?

2. **Adoption**
   a) How did you integrate the website into your clinical practice?
      - What strategies worked? Why do you think they worked?
      - What strategies didn't work? Why didn't they work? /did not?
      - To what degree did you promote the website? (e.g., frequency, assumed reach)

   b) Who do you feel this website is for?
      - Informal criteria for selecting patients; patient "readiness"

   c) Tell me about how you have introduced or integrated the website into clinical care?

3. **Implementation**
   a) Throughout the implementation we offered training and support to promote/engage patients with the website. Are you aware of some of these strategies? Tell me about how you have used these?
      - What do you like/not like? Why?
      - Have you sought additional resources?

   b) Have you provided the website to patients that do not have CKD? If yes, please describe those scenarios and why you provided the website.
      - Patients with diabetes, hypertension, other

4. **Maintenance**

a) What is the likelihood that you will continue to support this intervention (i.e., continue to promote the website)?

b) We are planning to work with other clinics within Alberta and outside Alberta. What would be needed for other clinicians/clinics to integrate this into clinical care?

- *Recommendations?*

c) Are you doing anything different in your practice to include the website in supporting your patients?

d) What measures would you be interested in to assess the impact of the website on your patients?
